# Supplementary material for: Knowledge, facilitators and barriers to cervical cancer screening among women in Uganda: a qualitative study
Source: BMJ Open. 2017 Jun 11;7(6):e016282. doi: 10.1136/bmjopen-2017-016282 (PMC5541520; doi:10.1136/bmjopen-2017-016282)
Supplement: Author's manuscript [file bmjopen-2017-016282.draft_revisions.pdf]

Knowledge, facilitators and barriers to cervical cancer screening among women in Uganda: a qualitative study

Journal:	*BMJ Open*
Manuscript ID	bmjopen-2017-016282
Article Type:	Research
Date Submitted by the Author:	08-Feb-2017
Complete List of Authors:	Ndejjo, Rawlance; Makerere University School of Public Health, Department of Disease Control and Environmental Health<br>Mukama, Trasias; Makerere University School of Public Health, Department of Disease Control and Environmental Health<br>Kiguli, Juliet; Makerere University School of Public Health, Department of Community Health and Behavioural Sciences<br>Musoke, David; Makerere University School of Public Health, Department of Disease Control and Environmental Health
**Primary Subject Heading**:	Oncology
Secondary Subject Heading:	Qualitative research, Public health
Keywords:	barriers, cervical cancer, facilitators, knowledge, Uganda, beliefs

SCHOLARONE™ Manuscripts

For peer review only - http://bmjopen.bmj.com/site/about/guidelines.xhtml

Knowledge, facilitators and barriers to cervical cancer screening among women in Uganda: a qualitative study

Rawlance Ndejjo[1], Trasias Mukama[1], Juliet Kiguli[2], David Musoke[1].

1. Department of Disease Control and Environmental Health, School of Public Health, College of Health Sciences, Makerere University, Kampala, Uganda.

2. Department of Community Health and Behavioural Sciences, School of Public Health, College of Health Sciences, Makerere University, Kampala, Uganda.

Correspondence to

Rawlance Ndejjo,
Department of Disease Control and Environmental Health, School of Public Health, College of Health Sciences, Makerere University. P.O. Box 7072, Kampala, Uganda.
Email: rndejjo@musph.ac.ug
Telephone: +256777783971

For peer review only

Word count: 4851

Number of tables: 1

Number of references: 28

Abstract

Objectives: To explore community knowledge, facilitators and barriers to cervical cancer screening among women in rural Uganda so as to generate data to inform interventions.

Design: A qualitative study utilizing focus group discussions and key informant interviews.

Setting: Discussions and interviews carried out in the community within the two districts in Uganda.

Participants: Ten (10) focus group discussions with 119 screening-eligible women aged between 25 to 49 years, and 11 key informant interviews with health care providers and administrators.

Results: Study participants' knowledge about cervical cancer causes, signs and symptoms, testing methods and prevention was poor. Many participants attributed the cause of cervical cancer to use of contraception while key informants said that some believed it was due to witchcraft. Perceptions towards cervical cancer and screening were majorly positive with many participants stating that they were at risk of getting cervical cancer. The facilitators to accessing cervical cancer screening were: experiencing signs and symptoms of cervical cancer, family history of the disease, and awareness of the disease / screening service. Lack of knowledge about cervical cancer and screening, health system challenges, fear of test outcome and consequences, and financial constraints were barriers to cervical cancer screening.

Conclusion: Whereas perceptions towards cervical cancer and screening were positive, knowledge of study participants on cervical cancer was poor. To improve cervical cancer screening, effort should be focused on reducing identified barriers and enhancing facilitators.

Keywords: barriers, beliefs, cervical cancer, facilitators, knowledge, Uganda,

For peer review only

Strengths and limitations of this study

This study involved focus group discussions with women and key informant interviews with health workers and administrators which enabled triangulation of responses thus increasing validity of the study.

Data collection took place in natural settings providing a conducive environment for women to openly express themselves and share experiences.

The study was carried out in two majorly rural districts and results may not be generalizable to the different settings.

Study findings have potential to influence design of future cervical cancer programs and services.

For peer review only

INTRODUCTION

Globally, in 2012, cervical cancer was responsible for 265,700 deaths and 527,600 diagnoses, 85% of which occurred in developing countries [1 2]. In East Africa, it is the leading cause of cancer deaths and has the highest age-standardised incidence rates of 42.7 per 100,000 women per year [1 2]. Estimates for Uganda show that cervical cancer led to 2,300 deaths and 4,000 new cases in 2012 [3]. Majority of these deaths are preventable through human papilloma virus (HPV) vaccination for young girls, and screening for pre-cancerous lesions for women at risk. In developed countries, where prevention and control interventions have been implemented, reductions in detected cases and deaths have been registered over the past three decades [4].

Cervical cancer screening rates in Uganda are very low despite the higher intention and willingness to screen. For example, a study conducted in central Uganda reported a screening rate of 7% [5] while another in the eastern part of the country reported 4.8% [6]. Low cervical cancer screening rates have also been reported among health workers in Uganda [7]. Moreover, most cancers diagnosed in Uganda are already in advanced stage, when no remedial action can be taken [8]. Community level factors such as: knowledge, attitudes and access to screening services can have implications for the successful implementation of screening programs and other public health interventions [6 9 10]. Specifically, factors such as a feeling of embarrassment, perceived pain during examination, fatalism associated with a diagnosis, unsupportive husbands, and lack of awareness about cervical cancer and available services can play a key role [5 11-13].

Strategies such as community mobilization and education, peer to peer engagement, and organizing health systems to track and follow up women have been suggested to mitigate barriers and optimize chances of success of screening programs [12 14]. To be effective, such interventions require a deeper understanding of the social contextual factors influencing uptake of cervical cancer screening services. The few studies that have explored factors affecting uptake of cervical cancer screening in the eastern African region have mainly been quantitative and not had a deep exploration of such factors to fully inform future interventions. Therefore, the purpose of this study was to explore community knowledge, facilitators and barriers to cervical cancer screening among women in rural Uganda so as to generate data to inform interventions.

For peer review only

METHODS

Study area, design and population

This cross sectional study was carried out in eastern Uganda in two majorly rural districts of Bugiri and Mayuge, which are approximately 150 kilometres from Kampala, the country's capital. These districts have a combined population of 856,152 of whom 51.4% are females [15], and occupy approximately 10,372 square km. The major economic activities in the districts include: subsistence agriculture, fishing and operation of small businesses in trading centres. The study involved focus group discussions (FGDs) with women aged between 25 and 49 years who had lived in the study districts for a minimum of six (6) months. This age group was chosen because of their eligibility to access screening and higher risk of cervical cancer [4]. Key informant interviews (KIIs) were also carried out with members of the district health teams including the district health officers, and health workers in both public and private health facilities at different levels such as nurses, medical officers, clinical officers and midwives.

Sampling

We randomly selected five sub counties in each district (out of nine from Bugiri and seven from Mayuge) and purposively chose one village from each from which the FGD participants were identified. Villages were chosen to facilitate a wide geographical coverage of the study area as well as sub-populations of women in terms of rural-urban residence and socio-economic status. One FGD was then conducted per selected village, making a total of 10 FGDs. Local leaders and community health workers in the selected villages guided the identification and recruitment of participants fulfilling the inclusion criteria. The participants were then approached and invited to participate in the FGDs. Participants of the KIIs were purposively selected basing on their technical involvement in decision making relating to cervical cancer screening services provision. Eleven (11) KIIs were conducted in the two districts. These numbers of FGDs and KIIs enabled a theoretical saturation, when no new ideas were emerging.

For peer review only

Data collection

Thematic guides for FGDs and KIIs, developed basing on previous literature and pretested among a similar study population, were used during data collection. The FGD guide explored knowledge and beliefs of women about cervical cancer, access to cervical cancer screening including barriers and facilitating factors, and recommendations to improve screening uptake among women. On the other hand, the KII guide had questions on community knowledge and perceptions about cervical cancer, access to screening services, capacity to carry out cervical cancer screening, and measures to increase utilization of screening services. Both guides had several probes and prompts to guide the Research Assistants during data collection. The FGDs, that had an average of 12 participants, were conducted by two trained female Research Assistants with vast experience in conducting qualitative research and native speakers of *Lusoga,* the main local language used in the study area. The Research Assistants were supervised by a member of the research team throughout the data collection process for quality control. One Research Assistant moderated and facilitated the discussions while the other assisted in taking notes and recording the interviews. FGDs were conducted in community gathering places identified by the research team in collaboration with local leaders. Sites for interviews were carefully selected to reduce interference from non-participants, and all study participants were encouraged to openly discuss their opinions. Local leaders arranged the venues, mobilized the participants, and agreed with them a convenient time for the discussion. FGDs lasted on average one hour, excluding 10 – 15 minutes that Research Assistants spent administering consent and building rapport. For KIIs, after key informants were identified, appointments were made by phone, and interviews scheduled at convenient times. The interviews were conducted by the researchers in places convenient to the informants mostly at their work place lasting an average of 45 minutes. All FGDs and KIIs were audio-recorded.

Data management and analysis

The labeled recordings of the discussions and interviews were fully transcribed verbatim from the local language to English and proof read several times by the Research Assistants. The transcripts were then read several times by all research team members and any emerging themes noted. Two researchers then developed the code book for data analysis and described the coding

For peer review only

tree, which was reviewed and discussed with other researchers and any differences harmonised. Data was coded after interpretation and ascribing meaning, and analysed using directed content analysis [16] with the help of Atlas ti version 6.0.15 qualitative data management software. From the analysis, direct quotations from the FGD participants and KIs are presented in italics to highlight and support key findings.

Ethical considerations

The study protocol was reviewed and approved by the Higher Degrees Research and Ethics Committee at Makerere University School of Public Health, and the study registered with the Uganda National Council for Science and Technology. Written informed consent was obtained from all participants after the moderator explained the study aims, benefits and potential risks. Participants' anonymity was maintained throughout the research process through use of numbers and confidential treatment of data.

For peer review only

RESULTS

Socio-demographic characteristics of participants

Among the 119 FGD participants, the mean age was 34.4 years (SD = ±7.0) and most (50; 42.0%) were aged between 31 to 40 years, married 93 (78.1%) and had attained primary as highest level of education 58 (48.7%). Most participants were peasant farmers 69 (58.0%) and majority 82 (68.8%) had lived in the area for up to 20 years (Table 1).

Table 1: Socio-demographic characteristics of participants

Characteristics	Frequency (n=119)	Percent (%)
**District**		
Bugiri	50	42.0
Mayuge	69	58.0
**Age (years)**	Mean = 34.4, SD = ±7.0	
25-30	47	39.5
31-40	50	42.0
40-49	22	18.5
**Marital status**		
Married	93	78.1
Not married	26	21.9
**Education level**		
None	6	5.0
Primary	58	48.7
Secondary	51	42.9
Tertiary/university	4	3.4
**Occupation**		
Peasant farmer	69	58.0
Business	22	18.5
House wife	16	13.4
Others (health worker, teacher)	12	10.1
**Duration in area (years)**	Mean = 16.2, SD= ±11.7	
Less than 10	41	34.4
Between 10 to 20	41	34.4
More than 20	37	31.1

For peer review only

Knowledge and beliefs about cervical cancer

This theme explored women's knowledge and awareness regarding causes, signs and symptoms, risk factors and screening methods for cervical cancer. In addition, it explored women's beliefs of being at risk of cervical cancer, and whether and how the disease can be prevented and treated.

Causes of cervical cancer

Almost all participants had heard about cervical cancer mainly from radios with the rest getting information from health workers at health facilities and a few from traditional health practitioners. However, knowledge about cervical cancer was poor and several misconceptions existed among participants. In fact, many participants across the different FGDs consistently stated that cervical cancer resulted from use of contraceptives. This notion was majorly related to the side effects of some contraception methods including excessive bleeding and effects on menstruation cycles.

"I say contraception causes cervical cancer because when you start using certain options like swallowing pills, you find yourself bleeding so much meaning they affect you a lot, you bleed and bleed without getting treatment." [FGD 7, Participant 9, 40 years]

Some attributed cervical cancer to the foods they ate and its preparation including use of polythene bags to cover food when cooking while others highlighted other factors such as: having either many or fewer children, long use / nonuse of sanitary pads, abortions, sharing hygiene facilities like bathrooms, and improper personal hygiene.

The key informants also confirmed the belief among women that cervical cancer was due to use of contraception methods.

"Many women state that they got cervical cancer due to use of contraceptives because it too is associated with bleeding. They forget that even before we started using contraception methods, cancer was in existence." [Health worker at private facility, Mayuge]

Although none of the participants said that cervical cancer was associated with witchcraft, several KIs highlighted this as a perception that existed among women in the community. This

For peer review only

they said also contributed to women seeking screening late as they could first go to the traditional health practitioners before interacting with the formal health system.

"The community is ignorant and associate cervical cancer with witchcraft. They say that 'a co-wife or someone else bewitched me so that is why I am bleeding'." [Health worker at government health facility, Bugiri]

Signs and symptoms of cervical cancer

Knowledge of signs and symptoms of cervical cancer was very poor and participants continuously stressed that they needed to be educated more to understand how the disease manifests.

"People don't even know the symptoms of cervical cancer for instance as one would know that a high fever and/or vomiting is for malaria but for cervical cancer we don't know the symptoms." [FGD 4, Participant 11, 40 years]

The few who knew some signs and symptoms of cervical cancer had either had a personal experience with the disease or with those affected by it. The major symptoms listed were vaginal bleeding, backache and abdominal pain.

"My aunt began with bleeding abnormally and yet she had stopped menstruation. This persisted and she started seeking for treatment with the hope of becoming well. After some time, they diagnosed her with late stage cervical cancer and told her it would not be cured." [FGD 9, Participant 8, 43 years]

Risk factors for cervical cancer

Knowledge about risk factors for cervical cancer was good as many participants noted that having: many sexual partners, sexually transmitted infections, and family history of cervical cancer would increase one's risk. This was also noted by some KIs.

For peer review only

"I think having many sexual partners could lead to cervical cancer because the men also get various infections like gonorrhea and some are even HIV positive. Therefore having many sexual partners could bring cancer to you." [FGD 4, Participant 13, 37 years]

Screening methods for cervical cancer

Knowledge about methods used for cervical cancer screening was poor with many participants stating that they had never heard of any method.

"We have never suffered from cervical cancer and neither have we screened for the disease. We thus don't know any methods used during screening." [FGD 10, participant 5, 45 years]

However, a few participants described the methods they had experienced or heard of from their peers.

"The method that I know is they make you lay on your back and there is an equipment they insert in your private parts so that the doctor examining you can see the opening of the cervix well and whether it has wounds or not." [FGD 5, Participant 2, 42 years]

"I heard that the equipment they use helps in getting a sample from the cervix which they test to find out whether you have cancer or not." [FGD 2, Participant 1, 30 years]

Perception of risk of cervical cancer

Almost all participants reported that they were at risk of getting cervical cancer giving several reasons ranging from their use of family planning methods to their lack of knowledge about signs and symptoms and preventive measures of the disease. They made reference to some risk factors of the disease such as cervical cancer being hereditary and possibility of acquiring it during sex which they said put them at risk. Many participants also perceived that cervical cancer was a big problem in their communities though could not estimate its prevalence.

"I think we are at risk because we don't know how this cancer is prevented and what we should use to avoid getting it so this lack of knowledge puts us at risk." [FGD 2, Participant 2, 32 years]

For peer review only

Prevention of cervical cancer

Most participants believed that cervical cancer could be prevented after understanding the causes of the disease, how it can be avoided and screening to know one's status.

"Preventing cervical cancer is possible after understanding its causes but before that, we still have a long way to go." [FGD 7, Participant 6, 26 years]

"I want to know that I don't have cervical cancer like I know that I don't have HIV/AIDs so that I can avoid it." [FGD 9, Participant 10, 25 years]

Treatment of cervical cancer and outcome

Asking whether cervical cancer could be treated, most participants agreed that something can be done when the cancer is in its early stages though stated that when there are delays to access treatment, the cancer would not be cured. Other participants stated otherwise.

"I think cervical cancer is treatable; if you get to know early, they test and treat you. On the other hand, when you seek care while it is already out of hand, it might not be cured." [FGD 10, Participant 10, 35 years]

Facilitators of cervical cancer screening

Among the study participants, only 5 (4.2%) had ever screened for cervical cancer and these were in only 3 of the 10 FGDs. This notwithstanding, most women were interested in receiving cervical cancer screening regularly and cited reasons such as wanting to: know their status, prevent cervical cancer and obtain treatment if they had the disease. The participants also stated that this willingness was shared by other women in the community. Study participants who had screened for the disease mostly had had its signs and symptoms. The other facilitators noted were: family history of cervical cancer and awareness of the disease / screening service.

For peer review only

Experiencing signs and symptoms of cervical cancer

The greatest facilitator of cervical cancer screening among the study participants was having experienced signs and symptoms of the disease which prompted them to visit health facilities for consultation. The manifestation of signs and symptoms also determined the number of times one went to the facility and some women pointed it out as an important determinant for accessing the screening service even in the future.

"I used to go for cervical cancer screening whenever I could over bleed. So I have so far screened thrice." [FGD 4, participant 1, 49 years]

"The reason I went for screening is because I used to get abdominal pain during my menstruation. Although I did not get my results, they gave me some drugs which I took and became fine. I have never gone back ever since." [FGD 2, respondent 10, 35 years]

Family history of cervical cancer

A few participants reported family history as a facilitator for accessing cervical cancer screening among women.

"I recently buried my sister because of this cancer and before she died her cervix had been removed. Following this, my mother was also screened and she was told that she had cervical cancer." [FGD 2, participant 5, 38 years]

Awareness of the disease / screening service

Some women had screened for cervical cancer because they had accessed information mainly through radios and health facilities regarding the disease and understood the importance of screening. Additionally, others knew about the availability of free screening services at some health facilities.

"There is a time they announced that there was free screening for cervical cancer at the health facility and I went there although I did not follow up on my results." [FGD 2, participant 10, 35 years]

For peer review only

Barriers to cervical cancer screening

When participants were asked about barriers affecting their uptake of cervical cancer screening, several sub-themes emerged: lack of knowledge about cervical cancer and screening, health system challenges, fear of test outcome and consequences, and financial constraints. These were in agreement with barriers highlighted by the key informants.

Lack of knowledge about cervical cancer and screening

Throughout the FGDs, lack of knowledge about cervical cancer and screening was continuously highlighted as a barrier to accessing the service. In addition, study participants stated that due to their lack of knowledge, it was sometimes hard for them to access screening services without relating their symptoms with the disease. The key informants also reechoed this stating that most women do not take the initiative to access screening without the signs and symptoms of the disease.

"We are constrained from accessing the service because by the time you get the cancer, you will not have known the symptoms early enough yet without having any symptoms, people are lazy to go for cervical cancer screening." [FGD 5, Participant 7, 45 years]

Health system challenges

The major health system challenges from the FGDs were the lack of health facilities offering screening, lack of awareness of services availability, services being far away from the community and the mistreatment of women by health workers at health facilities.

"From the mobilizations that we do, some people would like to undergo the screening but there is no nearby health facility that is offering the service. Then in the health facilities, some health workers are also rude to women and so some give up on going there and rather go to traditional herbalists for help." [FGD 2, Participant 8, 28 years]

For peer review only

The key informants mainly highlighted inadequate human resources, lack of proper training to carry out screening and lack of screening materials at health facilities as barriers to cervical cancer screening.

"We have 2 health facilities that offer the service but these do not have medical officers and the available clinical officers do not have the capacity to screen for cervical cancer thus limiting screening to only one major hospital." [Health worker at government facility, Bugiri]

Fear of test, outcome and consequences

Women expressed different concerns about the testing methods used during cervical cancer screening from the way they are handled by health workers to who handles them. They said they were uncomfortable undressing themselves before health workers especially if they were male. Some of these fears were attributed to their bad previous experience or that of their colleagues.

"There are doctors who come here once in a while and they call us for screening but they handle you like you are going to give birth. They roughly insert an equipment inside and you feel pain. Even if women come and they hear it's the same method, very few will go there." [FGD 9, Participant 3, 34 years]

".... the part that is affected by that cancer makes us uncomfortable when it comes to showing the health worker. This testing method should at least be improved" [FGD 5, Participant 13, 40 years]

The key informants also expressed these fears as barriers which they attributed to culture, male health workers, and significant age difference where older women are handled by younger health workers.

"You know cancers cut across all ages, a client of 40 years may come to a health worker of 20 years and would be like, "you are my daughter, how can I undress myself to you?" on the other hand, the youth would say 'how can I expose myself to my mother?'. So the attitude towards age difference becomes a problem. [Health worker at private facility, Mayuge]

For peer review only

Women also expressed fear of a positive diagnosis. Others noted that women could equally fear finding out their HIV status if this were to be provided during the visit. Participants also stated that testing for cervical cancer could lead to unfavourable consequences especially among women including being left by their male spouses if found positive or not being able to have more children.

"Women don't screen because they think that if their husbands know that they have cancer, they will look for other women who don't have it and leave them. Some men think that if a woman has cervical cancer she can spread it to them or even produce children with cancer. Men are also concerned about the resultant treatment expenses. [FGD 2, Participant 6, 40 years]

Financial constraints

Many women highlighted that they were also constrained by lack of finances to cater for transport to visit screening centres, screening costs especially when accessing service from private providers, and treatment costs if found to have the disease.

"Lack of money for transport prevents us from accessing the service; sometimes you don't know the place and you don't have anyone to help take you there and neither do you have the money to go with." [FGD 10, Participant 3, 37 years]

This was also noted by a KI who worked in a private health facility where there was a cost attached to accessing the service.

"I think people who come for cervical cancer screening will need money which they may not have. Although the service is free in government health facilities, sometimes the reagents are not there and so people don't want to go there. When they come to a private setting, there is a cost attached to the service which can affect utilization." [Health worker at private facility, Mayuge]

For peer review only

DISCUSSION

This study explored women's knowledge, facilitators and barriers to cervical cancer screening in rural Uganda. From the study, knowledge about cervical cancer causes, signs and symptoms, screening methods and prevention was poor. This is consistent with findings from another study carried out in Uganda [10]. Common misconceptions about cervical cancer included the use of contraceptives being perceived as a cause of the disease which has previously been reported and discussed by other studies [10 17 18]. The urgency for health education campaigns on cervical cancer among the population can therefore not be overemphasized. On the other hand, awareness of some cervical cancer risk factors was high and most participants perceived themselves to be at risk of the disease. Although some study participants justified this using held misconceptions, it is still positive for cervical cancer control programs. Moreover, risk perception is a critical factor in promoting precautionary health behavior and has been a determinant of cervical cancer screening in previous studies [7 13].

Although among the FGD participants, only 4 in 100 had been screened for cervical cancer, most participants showed interest in accessing the service more regularly. Such high willingness for cervical cancer screening has also been documented by previous research in Uganda [5 19] and elsewhere [13 20]. Facilitators for cervical cancer screening among study participants were: experiencing signs and symptoms of cervical cancer, having a family history of the disease and being aware of cervical cancer and screening services, similar to those reported by previous studies [6 21]. The belief among study participants that screening should be accessed after experiencing cervical cancer signs and symptoms is negative for the success of screening programs. This further reinforces the need to increase awareness about cervical cancer among women. In fact, the preference for screening being asymptomatic women, education campaigns should encourage women aged between 30 and 49 years to screen for cervical cancer at least once in their lifetime as recommended by the World Health Organization [4]. Since availability of services alone is not sufficient to facilitate screening, measures should be put in place to publicize such services to increase their uptake including through use of mass media such as radio and television. Community sensitizations and mobilization for cervical cancer programs can also be contributed to by community health workers, an important cadre in supporting health systems especially in developing countries [22 23]. Indeed, community health workers have significantly contributed to cervical cancer screening and prevention efforts previously [24 25].

For peer review only

In this study, barriers to cervical cancer screening reported were: lack of knowledge about cervical cancer and screening; health system challenges; fear of test, outcome and consequences; and financial constraints. These barriers are similar to those documented by previous researches [13 14 18 21 26]. To facilitate uptake of cervical cancer screening, sensitizations should be carried out to increase awareness about the disease and importance of screening. Also critical is the need increase access to cervical cancer screening services within communities to address health system challenges such as long distances to health facilities and transport costs. To achieve this in a developing country perspective, there is need to adopt a community outreach model of service delivery where screening services are extended to the community regularly while building capacity at lower health centres. Integration of cervical cancer screening services with others accessed by women such as antenatal care, family planning or post-natal care could also increase screening uptake. In their assessment among policy makers and the community of integration of HIV and cervical cancer screening services, Kumakech et al. highlighted several concerns [27 28]. These include: limited health system capacity, potential consequences of integration, prolonged waiting times at the health facility and tiredness among women and health workers. It is therefore important that such factors are also considered before any integration of cervical cancer screening with other services. In addition, since some women according to this study associated cervical cancer with use of contraceptives, such integration should be properly designed and implemented or else would run the risk of re-enforcing this myth as well as affecting uptake of both services.

The need for more female staff to carry out screening due to the embarrassment felt by some when attended to by male staff was also highlighted. This concern among women has been reported by many previous studies [14 18 21 26]. This gap can be bridged by building the capacity of available female health workers some of whom may be of lower cadres such as nurses and midwives through task shifting to carryout cervical cancer screening of women with support from other staff. It is also important to have a mix of both younger and older staff to cater for all clients. Additionally, the importance of having a well-functioning health system cannot be overstated in this context. In this, the health facilities should have adequate capacity including skilled and professional workforce and required supplies and logistics to provide quality screening services to women. These measures should have a positive impact on cervical cancer screening rates.

For peer review only

This study had strengths and limitations. Firstly, it solicited views from women and key informants using FGDs and KIIs respectively which allowed for varied responses and enabled triangulation of findings thus increasing the validity of the study. Secondly, data collection took place in natural settings providing a conducive environment for women to openly express themselves and share experiences. On the other hand, although the study was carried out in two majorly rural districts and results not be generalizable to the whole country, it reports important information that could be instrumental in the design of future cervical cancer programs and services.

CONCLUSIONS

Although perceptions towards cervical cancer and screening were positive, knowledge of study participants on cervical cancer was poor highlighting an urgent need to prioritize sensitization and provision of communities with adequate information about cervical cancer. To improve cervical cancer screening, effort should be focused on reducing identified barriers and enhancing facilitators through measures such as raising awareness about the disease, strengthening health systems capacity, and utilizing female health workers to carry out screening.

For peer review only

Acknowledgements The authors thank the study participants for their time and contribution to this study. Appreciation is also extended to the Research Assistants, Jackline Mwendeze and Prossie Aliwebwa for the support they provided during data collection.

Contributors RN, TM, JK and DM contributed to study design and implementation, and data analysis. RN and TM drafted the manuscript. DM and JK critically reviewed the manuscript. All authors read and approved the final version.

Competing interests None.

Funding This work was supported by Training Health Researchers into Vocational Excellence in East Africa (THRiVE), grant number 087540 funded by the Wellcome Trust. Its contents are solely the responsibility of the authors and do not necessarily represent the official views of the supporting offices. The funders had no role in study design, data collection and analysis, decision to publish, or preparation of the manuscript.

Ethics approval Higher Degrees Research and Ethics Committee at Makerere University School of Public Health

Data sharing statement No additional data are available.

For peer review only

References

1. Torre LA, Bray F, Siegel RL, et al. Global cancer statistics, 2012. *CA: a cancer journal for clinicians* 2015;65(2):87-108.
2. Ferlay J, Soerjomataram I, Dikshit R, et al. Cancer incidence and mortality worldwide: Sources, methods and major patterns in GLOBOCAN 2012. *International Journal of Cancer* 2015;136(5):E359-E86. doi: 10.1002/ijc.29210
3. Bruni L, Barrionuevo-Rosas L, Albero G, et al. Human Papillomavirus and Related Diseases in Uganda. Summary Report 2016: ICO Information Centre on HPV and Cancer (HPV Information Centre).
4. World Health Organization. Comprehensive Cervical Cancer Control: A Guide to Essential Practice. 2nd edition. Geneva: World Health Organization, 2014.
5. Twinomujuni C, Nuwaha F, Babirye JN. Understanding the Low Level of Cervical Cancer Screening in Masaka Uganda Using the ASE Model: A Community-Based Survey. *PloS one* 2015;10(6):e0128498.
6. Ndejjo R, Mukama T, Musabyimana A, et al. Uptake of Cervical Cancer Screening and Associated Factors among Women in Rural Uganda: A Cross Sectional Study. *PLoS ONE* 2016;11(2):e0149696. doi: 10.1371/journal.pone.0149696
7. Mutyaba T, Mmiro FA, Weiderpass E. Knowledge, attitudes and practices on cervical cancer screening among the medical workers of Mulago Hospital, Uganda. *BMC medical education* 2006;6(1):13.
8. Mwaka AD, Okello ES, Wabinga H, et al. Symptomatic presentation with cervical cancer in Uganda: a qualitative study assessing the pathways to diagnosis in a low-income country. *BMC women's health* 2015;15(1):1.
9. Mwaka AD, Okello ES, Kiguli J, et al. Understanding cervical cancer: an exploration of lay perceptions, beliefs and knowledge about cervical cancer among the Acholi in northern Uganda. *BMC women's health* 2014;14(1):1.
10. Mutyaba T, Faxelid E, Mirembe F, et al. Influences on uptake of reproductive health services in Nsangi community of Uganda and their implications for cervical cancer screening. *Reprod Health* 2007;4(4)
11. Paul P, Winkler JL, Bartolini RM, et al. Screen-and-treat approach to cervical cancer prevention using visual inspection with acetic acid and cryotherapy: experiences,

For peer review only

perceptions, and beliefs from demonstration projects in Peru, Uganda, and Vietnam. *The oncologist* 2013;18(12):1278-84.

12. Teng FF, Mitchell SM, Sekikubo M, et al. Understanding the role of embarrassment in gynaecological screening: a qualitative study from the ASPIRE cervical cancer screening project in Uganda. *BMJ open* 2014;4(4):e004783.
13. Were E, Nyaberi Z, Buziba N. Perceptions of risk and barriers to cervical cancer screening at Moi Teaching and Referral Hospital (MTRH), Eldoret, Kenya. *African health sciences* 2011;11(1)
14. Mwaka AD, Wabinga HR, Mayanja-Kizza H. Mind the gaps: a qualitative study of perceptions of healthcare professionals on challenges and proposed remedies for cervical cancer help-seeking in post conflict northern Uganda. *BMC family practice* 2013;14(1):1.
15. Uganda Bureau of Statistics. The National Population and Housing Census 2014 – Main Report

Kampala: Uganda Bureau of Statistics, 2016.

16. Hsieh H-F, Shannon SE. Three approaches to qualitative content analysis. *Qualitative health research* 2005;15(9):1277-88.
17. Mukama T, Ndejjo R, Musabyimana A, et al. Women's knowledge and attitudes towards cervical cancer prevention: a cross sectional study in Eastern Uganda. *BMC Women's Health* 2017;17(1):9. doi: 10.1186/s12905-017-0365-3
18. Hasahya OT, Berggren V, Sematimba D, et al. Beliefs, perceptions and health-seeking behaviours in relation to cervical cancer: a qualitative study among women in Uganda following completion of an HPV vaccination campaign. *Global health action* 2016;9
19. Ndejjo R, Mukama T, Musinguzi G, et al. Women's intention to screen and willingness to vaccinate their daughters against cervical cancer – A cross sectional study in eastern Uganda. *BMC Public Health* 2016
20. Mingo AM, Panozzo CA, DiAngi YT, et al. Cervical cancer awareness and screening in Botswana. *International journal of gynecological cancer: official journal of the International Gynecological Cancer Society* 2012;22(4):638.
21. Ngugi CW, Boga H, Muigai AW, et al. Factors affecting uptake of cervical cancer early detection measures among women in Thika, Kenya. *Health care for women international* 2012;33(7):595-613.

For peer review only

22. Singh P, Sachs JD. 1 million community health workers in sub-Saharan Africa by 2015. *The Lancet* 2013;382(9889):363-65. doi: 10.1016/S0140-6736(12)62002-9

23. Bhutta ZA, Lassi ZS, Pariyo G, et al. Global experience of community health workers for delivery of health related millennium development goals: a systematic review, country case studies, and recommendations for integration into national health systems. *Global Health Workforce Alliance* 2010;1:249-61.

24. Elliott PF, Belinson SE, Ottolenghi E, et al. Community health workers, social support and cervical cancer screening among high-risk groups in rural Mexico. *Journal of health care for the poor and underserved* 2013;24(4):1448-59.

25. Agurto I, Arrossi S, White S, et al. Involving the community in cervical cancer prevention programs. *International Journal of Gynecology & Obstetrics* 2005;89:S38-S45.

26. Bukirwa A, Mutyoba JN, N.Mukasa B, et al. Motivations and barriers to cervical cancer screening among HIV infected women in HIV care: a qualitative study. *BMC Women's Health* 2015;15(1):1-11. doi: 10.1186/s12905-015-0243-9

27. Kumakech E, Andersson S, Wabinga H, et al. Integration of HIV and cervical cancer screening perceptions and preferences of communities in Uganda. *BMC women's health* 2015;15(1):1.

28. Kumakech E, Andersson S, Wabinga H, et al. Integration of HIV and cervical cancer screening perceptions of healthcare providers and policy makers in Uganda. *BMC public health* 2014;14(1):1.

For peer review only

BMJ Open

Knowledge, facilitators and barriers to cervical cancer screening among women in Uganda: a qualitative study

Journal:	*BMJ Open*
Manuscript ID	bmjopen-2017-016282.R1
Article Type:	Research
Date Submitted by the Author:	18-Apr-2017
Complete List of Authors:	Ndejjo, Rawlance; Makerere University School of Public Health, Department of Disease Control and Environmental Health<br>Mukama, Trasias; Makerere University School of Public Health, Department of Disease Control and Environmental Health<br>Kiguli, Juliet; Makerere University School of Public Health, Department of Community Health and Behavioural Sciences<br>Musoke, David; Makerere University School of Public Health, Department of Disease Control and Environmental Health
**Primary Subject Heading**:	Oncology
Secondary Subject Heading:	Qualitative research, Public health
Keywords:	barriers, cervical cancer, facilitators, knowledge, Uganda, beliefs

SCHOLARONE™ Manuscripts

Knowledge, facilitators and barriers to cervical cancer screening among women in Uganda: a qualitative study

Rawlance Ndejjo[1], Trasias Mukama[1], Juliet Kiguli[2], David Musoke[1].

1. Department of Disease Control and Environmental Health, School of Public Health, College of Health Sciences, Makerere University, Kampala, Uganda.

2. Department of Community Health and Behavioural Sciences, School of Public Health, College of Health Sciences, Makerere University, Kampala, Uganda.

Correspondence to

Rawlance Ndejjo,
Department of Disease Control and Environmental Health, School of Public Health, College of Health Sciences, Makerere University. P.O. Box 7072, Kampala, Uganda.
Email: rndejjo@musph.ac.ug
Telephone: +256777783971

Word count: 4937

Number of tables: 1

Number of references: 29

For peer review only - http://bmjopen.bmj.com/site/about/guidelines.xhtml

Abstract

Objectives: To explore community knowledge, facilitators and barriers to cervical cancer screening among women in rural Uganda so as to generate data to inform interventions.

Design: A qualitative study utilizing focus group discussions and key informant interviews.

Setting: Discussions and interviews carried out in the community within two districts in Eastern Uganda.

Participants: Ten (10) focus group discussions with 119 screening-eligible women aged between 25 to 49 years, and 11 key informant interviews with health care providers and administrators.

Results: Study participants' knowledge about cervical cancer causes, signs and symptoms, testing methods and prevention was poor. Many participants attributed the cause of cervical cancer to use of contraception while key informants said that some believed it was due to witchcraft. Perceptions towards cervical cancer and screening were majorly positive with many participants stating that they were at risk of getting cervical cancer. The facilitators to accessing cervical cancer screening were: experiencing signs and symptoms of cervical cancer, family history of the disease, and awareness of the disease / screening service. Lack of knowledge about cervical cancer and screening, health system challenges, fear of test outcome and consequences, and financial constraints were barriers to cervical cancer screening.

Conclusion: Whereas perceptions towards cervical cancer and screening were positive, knowledge of study participants on cervical cancer was poor. To improve cervical cancer screening, effort should be focused on reducing identified barriers and enhancing facilitators.

Keywords: barriers, beliefs, cervical cancer, facilitators, knowledge, Uganda,

For peer review only - http://bmjopen.bmj.com/site/about/guidelines.xhtml

Strengths and limitations of this study

- This study involved focus group discussions with women and key informant interviews with health workers and administrators which enabled triangulation of responses thus increasing validity of the study.
- Data collection took place in natural settings providing a conducive environment for women to openly express themselves and share experiences.
- The study was carried out in two majorly rural districts and results may not be generalizable to the different settings.
- Study findings have potential to influence design of future cervical cancer programs and services.

For peer review only

For peer review only - http://bmjopen.bmj.com/site/about/guidelines.xhtml

INTRODUCTION

Globally, in 2012, cervical cancer was responsible for 265,700 deaths and 527,600 diagnoses, 85% of which occurred in developing countries [1 2]. In East Africa, it is the leading cause of cancer deaths and has the highest age-standardised incidence rates of 42.7 per 100,000 women per year [1 2]. Estimates for Uganda show that cervical cancer led to 2,300 deaths and 4,000 new cases in 2012 [3]. Majority of these deaths are preventable through human papilloma virus (HPV) vaccination for young girls, and screening for pre-cancerous lesions for women at risk. In developed countries, where prevention and control interventions have been implemented, reductions in detected cases and deaths have been registered over the past three decades [4].

Cervical cancer screening rates in Uganda are very low despite the higher intention and willingness to screen. For example, a study conducted in central Uganda reported a screening rate of 7% [5] while another in the eastern part of the country reported 4.8% [6]. Low cervical cancer screening rates have also been reported among health workers in Uganda [7]. Moreover, most cancers diagnosed in Uganda are already in advanced stage, when no remedial action can be taken [8]. Community level factors such as: knowledge, attitudes and access to screening services can have implications for the successful implementation of screening programs and other public health interventions [6 9 10]. Specifically, factors such as a feeling of embarrassment, perceived pain during examination, fatalism associated with a diagnosis, unsupportive husbands, and lack of awareness about cervical cancer and available services can play a key role [5 11-13].

Strategies such as community mobilization and education, peer to peer engagement, and organizing health systems to track and follow up women have been suggested to mitigate barriers and optimize chances of success of screening programs [12 14]. To be effective, such interventions require a deeper understanding of the social contextual factors influencing uptake of cervical cancer screening services. The few studies that have explored factors affecting uptake of cervical cancer screening in the east African region have mainly been quantitative and not had a deep exploration of such factors to fully inform future interventions. Therefore, the purpose of this study was to explore community knowledge, facilitators and barriers to cervical cancer screening among women in rural Uganda so as to generate data to inform interventions.

For peer review only - http://bmjopen.bmj.com/site/about/guidelines.xhtml

METHODS

Study area, design and population

This cross sectional study was carried out in eastern Uganda in two majorly rural districts of Bugiri and Mayuge, which are approximately 150 kilometres from Kampala, the country's capital. These districts have a combined population of 856,152 of whom 51.4% are females [15], and occupy approximately 10,372 square km. The major economic activities in the districts include: subsistence agriculture, fishing and operation of small businesses in trading centres. The study involved focus group discussions (FGDs) with women aged between 25 and 49 years who had lived in the study districts for a minimum of six (6) months. This age group was chosen because of their eligibility to access screening and higher risk of cervical cancer [4]. Key informant interviews (KIIs) were also carried out with members of the district health teams including the district health officers, and health workers in both public and private health facilities at different levels such as nurses, medical officers, clinical officers and midwives.

Sampling

We randomly selected five sub counties in each district (out of nine from Bugiri and seven from Mayuge) and purposively chose one village from each from which the FGD participants were identified. Villages were chosen to facilitate a wide geographical coverage of the study area as well as sub-populations of women in terms of rural-urban residence and socio-economic status. One FGD was then conducted per selected village, making a total of 10 FGDs. Local leaders and community health workers in the selected villages guided the identification and purposive recruitment of participants fulfilling the inclusion criteria. The participants were then approached and invited to participate in the FGDs. Participants of the KIIs were purposively selected basing on their technical involvement in decision making relating to cervical cancer screening services provision. Eleven (11) KIIs were conducted in the two districts. These numbers of FGDs and KIIs enabled a theoretical saturation, when no new ideas were emerging.

For peer review only - http://bmjopen.bmj.com/site/about/guidelines.xhtml

Data collection

Thematic guides for FGDs and KIIs, developed basing on previous literature and pretested among a similar study population, were used during data collection which took two weeks. The FGD guide explored knowledge and beliefs of women about cervical cancer, access to cervical cancer screening including barriers and facilitating factors, and recommendations to improve screening uptake among women. On the other hand, the KII guide had questions on community knowledge and perceptions about cervical cancer, access to screening services, health system capacity to carry out cervical cancer screening, and opinion on measures to increase utilization of screening services. Both guides had several probes and prompts to guide the Research Assistants during data collection. The FGDs, that had an average of 12 participants, were conducted by two trained female Research Assistants with vast experience in conducting qualitative research and native speakers of *Lusoga,* the main local language used in the study area. No Research Assistants had any relationship with the study participants before the start of the study. The Research Assistants were supervised by a member of the research team throughout the data collection process for quality control. One Research Assistant moderated and facilitated the discussions while the other assisted in taking notes and recording the interviews. FGDs were conducted in community gathering places identified by the research team in collaboration with local leaders. Sites for interviews were carefully selected to reduce interference from non-participants, and all study participants were encouraged to openly discuss their opinions. Local leaders arranged the venues, mobilized the participants, and agreed with them a convenient time for the discussion. FGDs lasted on average one hour, excluding 10 – 15 minutes that Research Assistants spent administering consent and building rapport. For KIIs, after key informants were identified, appointments were made by phone, and interviews scheduled at convenient times. The interviews were conducted by the researchers in places convenient to the informants mostly at their work place lasting an average of 45 minutes. All FGDs and KIIs were audio-recorded.

Data management and analysis

The labeled recordings of the discussions and interviews were fully transcribed verbatim from the local language to English and proof read several times by the Research Assistants. The transcripts were then read several times by all research team members for familiarity with data

For peer review only - http://bmjopen.bmj.com/site/about/guidelines.xhtml

and any emerging themes noted in addition to others identified in advance. All researchers, RN (Environmental Health Scientist), TM (Public Health Specialist), JK (Anthropologist) and DM (Public Health Specialist) have experience in designing and conducting qualitative research. Three of the researchers (RN, TM and DM) are males while JK is female. Two researchers (RN and TM) independently developed the code book for data analysis and described the coding tree, which were then reviewed and discussed with other researchers and any differences harmonised. Data was coded after interpretation and ascribing meaning, and analysed using directed content analysis [16] with the help of Atlas ti version 6.0.15 qualitative data management software. From the analysis, direct quotations from the FGD participants and KIs are presented in italics to highlight and support key findings. The Consolidated criteria for reporting qualitative research (COREQ-32) checklist [17] guided the reporting of this study.

Ethical considerations

The study protocol was reviewed and approved by the Higher Degrees Research and Ethics Committee at Makerere University School of Public Health, and the study registered with the Uganda National Council for Science and Technology. Written informed consent was obtained from all participants after the moderator explained the study aims, benefits and potential risks. Participants' anonymity was maintained throughout the research process through use of numbers and confidential treatment of data.

For peer review only

For peer review only - http://bmjopen.bmj.com/site/about/guidelines.xhtml

RESULTS

Socio-demographic characteristics of participants

All participants approached to take part in the FGDs agreed to do so. Among the 119 FGD participants, the mean age was 34.4 years (SD = ±7.0) and most (50; 42.0%) were aged between 31 to 40 years, married 93 (78.1%) and had attained primary as highest level of education 58 (48.7%). Most participants were peasant farmers 69 (58.0%) and majority 82 (68.8%) had lived in the area for up to 20 years (Table 1).

Table 1: Socio-demographic characteristics of participants

Characteristics	Frequency (n=119)	Percent (%)
**District**		
Bugiri	50	42.0
Mayuge	69	58.0
**Age (years)**	Mean = 34.4, SD = ±7.0	
25-30	47	39.5
31-40	50	42.0
40-49	22	18.5
**Marital status**		
Married	93	78.1
Not married	26	21.9
**Education level**		
None	6	5.0
Primary	58	48.7
Secondary	51	42.9
Tertiary/university	4	3.4
**Occupation**		
Peasant farmer	69	58.0
Business	22	18.5
House wife	16	13.4
Others (health worker, teacher)	12	10.1
**Duration in area (years)**	Mean = 16.2, SD= ±11.7	
Less than 10	41	34.4
Between 10 to 20	41	34.4
More than 20	37	31.1

For peer review only - http://bmjopen.bmj.com/site/about/guidelines.xhtml

Knowledge and beliefs about cervical cancer

This theme explored women's knowledge and awareness regarding causes, signs and symptoms, risk factors and screening methods for cervical cancer. In addition, it explored women's beliefs of being at risk of cervical cancer, and whether and how the disease can be prevented and treated.

Causes of cervical cancer

Almost all participants had heard about cervical cancer mainly from radios with the rest getting information from health workers at health facilities and a few from traditional health practitioners. However, knowledge about cervical cancer was poor and several misconceptions existed among participants. In fact, many participants across the different FGDs consistently stated that cervical cancer resulted from use of contraceptives. This notion was majorly related to the side effects of some contraception methods including excessive bleeding and effects on menstruation cycles.

"I say contraception causes cervical cancer because when you start using certain options like swallowing pills, you find yourself bleeding so much meaning they affect you a lot, you bleed and bleed without getting treatment." [FGD 7, Participant 9, 40 years]

Some attributed cervical cancer to the foods they ate and its preparation including use of polythene bags to cover food when cooking while others highlighted other factors such as: having either many or fewer children, long use / nonuse of sanitary pads, abortions, sharing hygiene facilities like bathrooms, and improper personal hygiene.

The key informants also confirmed the belief among women that cervical cancer was due to use of contraception methods.

"Many women state that they got cervical cancer due to use of contraceptives because it too is associated with bleeding. They forget that even before we started using contraceptives, cancer was in existence." [Health worker at private facility, Mayuge]

Although none of the participants said that cervical cancer was associated with witchcraft, several KIs highlighted this as a perception that existed among women in the community. This

For peer review only - http://bmjopen.bmj.com/site/about/guidelines.xhtml

they said also contributed to women seeking screening late as they could first go to the traditional health practitioners before interacting with the formal health system.

"The community is ignorant and associate cervical cancer with witchcraft. They say that 'a co-wife or someone else bewitched me so that is why I am bleeding'." [Health worker at government health facility, Bugiri]

Signs and symptoms of cervical cancer

Knowledge of signs and symptoms of cervical cancer was very poor and participants continuously stressed that they needed to be educated more to understand how the disease manifests.

"People don't even know the symptoms of cervical cancer for instance as one would know that a high fever and/or vomiting is associated with malaria but for cervical cancer we don't know the symptoms." [FGD 4, Participant 11, 40 years]

The few who knew some signs and symptoms of cervical cancer had either had a personal experience with the disease or with those affected by it. The major symptoms they listed were vaginal bleeding, backache and abdominal pain.

"My aunt began with bleeding abnormally and yet she had stopped menstruation. This persisted and she started seeking for treatment with the hope of becoming well. After some time, they diagnosed her with late stage cervical cancer and told her it would not be cured." [FGD 9, Participant 8, 43 years]

Risk factors for cervical cancer

Knowledge about risk factors for cervical cancer was good as many participants noted that having: many sexual partners, sexually transmitted infections, and family history of cervical cancer would increase one's risk. This was also noted by some KIs.

For peer review only - http://bmjopen.bmj.com/site/about/guidelines.xhtml

"I think having many sexual partners could lead to cervical cancer because the men also get various infections like gonorrhea and some are even HIV positive. Therefore having many sexual partners could bring cancer to you." [FGD 4, Participant 13, 37 years]

Screening methods for cervical cancer

Knowledge about methods used for cervical cancer screening was poor with many participants stating that they had never heard of any method.

"We have never suffered from cervical cancer and neither have we screened for the disease. We thus don't know any methods used during screening." [FGD 10, participant 5, 45 years]

However, a few participants described the methods they had experienced or heard of from their peers.

"The method that I know is they make you lay on your back and there is an equipment they insert in your private parts so that the doctor examining you can see the opening of the cervix well and whether it has wounds or not." [FGD 5, Participant 2, 42 years]

"I heard that the equipment they use helps in getting a sample from the cervix which they test to find out whether you have cancer or not." [FGD 2, Participant 1, 30 years]

Perception of risk of cervical cancer

Almost all participants reported that they were at risk of getting cervical cancer giving several reasons ranging from their use of family planning methods to their lack of knowledge about signs and symptoms and preventive measures of the disease. They made reference to some risk factors of the disease such as cervical cancer being hereditary and possibility of acquiring it during sex which they said put them at risk. Many participants also perceived that cervical cancer was a big problem in their communities though could not estimate its prevalence.

"I think we are at risk because we don't know how this cancer is prevented and what we should use to avoid getting it so this lack of knowledge puts us at risk." [FGD 2, Participant 2, 32 years]

For peer review only - http://bmjopen.bmj.com/site/about/guidelines.xhtml

Prevention of cervical cancer

Most participants believed that cervical cancer could be prevented after understanding the causes of the disease, how it can be avoided and screening to know one's status.

"Preventing cervical cancer is possible after understanding its causes but before that, we still have a long way to go." [FGD 7, Participant 6, 26 years]

"I want to know that I don't have cervical cancer like I know that I don't have HIV/AIDs so that I can avoid it." [FGD 9, Participant 10, 25 years]

Treatment of cervical cancer and outcome

Asking whether cervical cancer could be treated, most participants agreed that something can be done when the cancer is in its early stages though stated that when there are delays to access treatment, the cancer would not be cured. Other participants stated otherwise.

"I think cervical cancer is treatable; if you get to know early, they test and treat you. On the other hand, when you seek care while it is already out of hand, it might not be cured." [FGD 10, Participant 10, 35 years]

Facilitators of cervical cancer screening

Among the study participants, only 5 (4.2%) had ever screened for cervical cancer and these were in only 3 of the 10 FGDs. This notwithstanding, most women were interested in receiving cervical cancer screening regularly and cited reasons such as wanting to: know their status, prevent cervical cancer and obtain treatment if they had the disease. The participants also stated that this willingness was shared by other women in the community. Study participants who had screened for the disease mostly had had its signs and symptoms. The other facilitators noted were: family history of cervical cancer and awareness of the disease / screening service.

For peer review only - http://bmjopen.bmj.com/site/about/guidelines.xhtml

Experiencing signs and symptoms of cervical cancer

The greatest facilitator of cervical cancer screening among the study participants was having experienced signs and symptoms of the disease which prompted them to visit health facilities for consultation. The manifestation of signs and symptoms also determined the number of times one went to the facility and some women pointed it out as an important determinant for accessing the screening service even in the future.

"I used to go for cervical cancer screening whenever I could over bleed. So I have so far screened thrice." [FGD 4, participant 1, 49 years]

"The reason I went for screening is because I used to get abdominal pain during my menstruation. Although I did not get my results, they gave me some drugs which I took and became fine. I have never gone back ever since." [FGD 2, respondent 10, 35 years]

Family history of cervical cancer

A few participants reported family history as a facilitator for accessing cervical cancer screening among women.

"I recently buried my sister because of this cancer and before she died her cervix had been removed. Following this, my mother was also screened and she was told that she had cervical cancer." [FGD 2, participant 5, 38 years]

Awareness of the disease / screening service

Some women had screened for cervical cancer because they had accessed information mainly through radios and health facilities regarding the disease and understood the importance of screening. Additionally, others knew about the availability of free screening services at some health facilities.

"There is a time they announced that there was free screening for cervical cancer at the health facility and I went there although I did not follow up on my results." [FGD 2, participant 10, 35 years]

For peer review only - http://bmjopen.bmj.com/site/about/guidelines.xhtml

Barriers to cervical cancer screening

When participants were asked about barriers affecting their uptake of cervical cancer screening, several sub-themes emerged: lack of knowledge about cervical cancer and screening, health system challenges, fear of test outcome and consequences, and financial constraints. These were in agreement with barriers highlighted by the key informants.

Lack of knowledge about cervical cancer and screening

Throughout the FGDs, lack of knowledge about cervical cancer and screening was continuously highlighted as a barrier to accessing the service. In addition, study participants stated that due to their lack of knowledge, it was sometimes hard for them to access screening services without relating their symptoms with the disease. The key informants also reechoed this stating that most women do not take the initiative to access screening without the signs and symptoms of the disease.

"We are constrained from accessing the service because by the time you get the cancer, you will not have known the symptoms early enough yet without having any symptoms, people are lazy to go for cervical cancer screening." [FGD 5, Participant 7, 45 years]

Health system challenges

The major health system challenges from the FGDs were the lack of health facilities offering screening, lack of awareness of services availability, services being far away from the community and the mistreatment of women by health workers at health facilities.

"From the mobilizations that we do, some people would like to undergo the screening but there is no nearby health facility that is offering the service. Then in the health facilities, some health

For peer review only - http://bmjopen.bmj.com/site/about/guidelines.xhtml

workers are also rude to women and so some give up on going there and rather go to traditional herbalists for help." [FGD 2, Participant 8, 28 years]

The key informants mainly highlighted inadequate human resources, lack of proper training to carry out screening and lack of screening materials at health facilities as barriers to cervical cancer screening.

"We have 2 health facilities that offer the service but these do not have medical officers and the available clinical officers do not have the capacity to screen for cervical cancer thus limiting screening to only one major hospital." [Health worker at government facility, Bugiri]

Fear of test, outcome and consequences

Women expressed different concerns about the testing methods used during cervical cancer screening from the way they are handled by health workers to who handles them. They said they were uncomfortable undressing themselves before health workers especially if they were male. Some of these fears were attributed to their bad previous experience or that of their colleagues.

"There are doctors who come here once in a while and they call us for screening but they handle you like you are going to give birth. They roughly insert an equipment inside and you feel pain. Even if women come and they hear it's the same method, very few will go there." [FGD 9, Participant 3, 34 years]

".... the part that is affected by that cancer makes us uncomfortable when it comes to showing the health worker. This testing method should at least be improved" [FGD 5, Participant 13, 40 years]

The key informants also expressed these fears as barriers which they attributed to culture, male health workers, and significant age difference where older women are handled by younger health workers.

"You know cancers cut across all ages, a client of 40 years may come to a health worker of 20 years and would be like, 'you are my daughter, how can I undress myself to you?' on the other hand, the youth would say 'how can I expose myself to my mother?'. So the attitude towards age difference becomes a problem. [Health worker at private facility, Mayuge]

For peer review only - http://bmjopen.bmj.com/site/about/guidelines.xhtml

Women also expressed fear of a positive diagnosis. Others noted that women could equally fear finding out their HIV status if this were to be provided during the visit. Participants also stated that testing for cervical cancer could lead to unfavourable consequences especially among women including being left by their male spouses if found positive or not being able to have more children.

"Women don't screen because they think that if their husbands know that they have cancer, they will look for other women who don't have it and leave them. Some men think that if a woman has cervical cancer she can spread it to them or even produce children with cancer. Men are also concerned about the resultant treatment expenses. [FGD 2, Participant 6, 40 years]

Financial constraints

Many women highlighted that they were also constrained by lack of finances to cater for transport to visit screening centres, screening costs especially when accessing service from private providers, and treatment costs if found to have the disease.

"Lack of money for transport prevents us from accessing the service; sometimes you don't know the place and you don't have anyone to help take you there and neither do you have the money to go with." [FGD 10, Participant 3, 37 years]

This was also noted by a KI who worked in a private health facility where there was a cost attached to accessing the service.

"I think people who come for cervical cancer screening will need money which they may not have. Although the service is free in government health facilities, sometimes the reagents are not there and so people don't want to go there. When they come to a private setting, there is a cost attached to the service which can affect utilization." [Health worker at private facility, Mayuge]

For peer review only - http://bmjopen.bmj.com/site/about/guidelines.xhtml

DISCUSSION

This study explored women's knowledge, facilitators and barriers to cervical cancer screening in rural Uganda. From the study, knowledge about cervical cancer causes, signs and symptoms, screening methods and prevention was poor. This is consistent with findings from another study carried out in Uganda [10]. Common misconceptions about cervical cancer included the use of contraceptives being perceived as a cause of the disease which has previously been reported and discussed by other studies [10 18 19]. The urgency for health education campaigns on cervical cancer among the population can therefore not be overemphasized. On the other hand, awareness of some cervical cancer risk factors was high and most participants perceived themselves to be at risk of the disease. Although some study participants justified this using held misconceptions such as their use of contraceptives, it is still positive for cervical cancer control programs. Moreover, risk perception is a critical factor in promoting precautionary health behavior and has been a determinant of cervical cancer screening in previous studies [7 13].

Although among the FGD participants, only 4 in 100 had been screened for cervical cancer, most participants showed interest in accessing the service more regularly. Such high willingness for cervical cancer screening has also been documented by previous research in Uganda [5 20] and elsewhere [13 21]. Facilitators for cervical cancer screening among study participants were: experiencing signs and symptoms of cervical cancer, having a family history of the disease and being aware of cervical cancer and screening services, similar to those reported by previous studies [6 22]. The belief among study participants that screening should be accessed after experiencing cervical cancer signs and symptoms is negative for the success of screening programs. This further reinforces the need to increase awareness about cervical cancer among women. In fact, the preference for screening being asymptomatic women, education campaigns should encourage women aged between 30 and 49 years to screen for cervical cancer at least once in their lifetime as recommended by the World Health Organization [4]. Since availability of services alone is not sufficient to facilitate screening, measures should be put in place to publicize such services to increase their uptake including through use of mass media such as radio and television. Community sensitizations and mobilization for cervical cancer programs can also be contributed to by community health workers, an important cadre in supporting health systems especially in developing countries [23 24]. Indeed, community health workers have significantly contributed to cervical cancer screening and prevention efforts previously [25 26].

For peer review only - http://bmjopen.bmj.com/site/about/guidelines.xhtml

In this study, barriers to cervical cancer screening reported were: lack of knowledge about cervical cancer and screening; health system challenges; fear of test, outcome and consequences; and financial constraints. These barriers are similar to those documented by previous researches [13 14 19 22 27]. To facilitate uptake of cervical cancer screening, sensitizations should be carried out to increase awareness about the disease and importance of screening. Also critical is the need increase access to cervical cancer screening services within communities to address health system challenges such as long distances to health facilities and transport costs. To achieve this in a developing country perspective, there is need to adopt a community outreach model of service delivery where screening services are extended to the community regularly while building capacity at lower health centres. Integration of cervical cancer screening services with others accessed by women such as antenatal care, family planning or post-natal care could also increase screening uptake. In their assessment among policy makers and the community of integration of HIV and cervical cancer screening services, Kumakech et al. highlighted several concerns [28 29]. These include: limited health system capacity, potential consequences of integration, prolonged waiting times at the health facility and tiredness among women and health workers. It is therefore important that such factors are also considered before any integration of cervical cancer screening with other services. In addition, since some women according to this study associated cervical cancer with use of contraceptives, such integration should be properly designed and implemented or else would run the risk of re-enforcing this myth as well as affecting uptake of both services.

The need for more female staff to carry out screening due to the embarrassment felt by some when attended to by male staff was also highlighted. This concern among women has been reported by many previous studies [14 19 22 27]. This gap can be bridged by building the capacity of available female health workers some of whom may be of lower cadres such as nurses and midwives through task shifting to carryout cervical cancer screening of women with support from other staff. It is also important to have a mix of both younger and older staff to cater for all clients. Additionally, the importance of having a well-functioning health system cannot be overstated in this context. In this, the health facilities should have adequate capacity including skilled and professional workforce and required supplies and logistics to provide quality screening services to women. These measures should have a positive impact on cervical cancer screening rates.

For peer review only - http://bmjopen.bmj.com/site/about/guidelines.xhtml

This study had strengths and limitations. Firstly, it solicited views from women and key informants using FGDs and KIIs respectively which allowed for varied responses and enabled triangulation of findings thus increasing the validity of the study. Secondly, data collection took place in natural settings providing a conducive environment for women to openly express themselves and share experiences. On the other hand, the FGD environment could have influenced participants to give answers that they perceive to be more socially acceptable. Also, during FGDs, sometimes there is dominance by outspoken participants which was minimized by having experienced moderators who ensured that all participants are involved in the discussion. Lastly, although the study was carried out in two majorly rural districts and results not be generalizable to the whole country, it reports important information that could be instrumental in the design of future cervical cancer programs and services.

CONCLUSIONS

Although perceptions towards cervical cancer and screening were positive, knowledge of study participants on cervical cancer was poor highlighting an urgent need to prioritize sensitization and provision of communities with adequate information about cervical cancer. To improve cervical cancer screening, effort should be focused on reducing identified barriers and enhancing facilitators through measures such as raising awareness about the disease, strengthening health systems capacity, and utilizing female health workers to carry out screening.

For peer review only - http://bmjopen.bmj.com/site/about/guidelines.xhtml

Acknowledgements The authors thank the study participants for their time and contribution to this study. Appreciation is also extended to the Research Assistants, Jackline Mwendeze and Prossie Aliwebwa for the support they provided during data collection.

Contributors RN, TM, JK and DM contributed to study design and implementation, and data analysis. RN and TM drafted the manuscript. DM and JK critically reviewed the manuscript. All authors read and approved the final version.

Competing interests None.

Funding This work was supported by Training Health Researchers into Vocational Excellence in East Africa (THRiVE), grant number 087540 funded by the Wellcome Trust. Its contents are solely the responsibility of the authors and do not necessarily represent the official views of the supporting offices. The funders had no role in study design, data collection and analysis, decision to publish, or preparation of the manuscript.

Ethics approval Higher Degrees Research and Ethics Committee at Makerere University School of Public Health

Data sharing statement No additional data are available.

For peer review only

References

1. Torre LA, Bray F, Siegel RL, Ferlay J, Lortet□Tieulent J, Jemal A: Global cancer statistics, 2012. *CA: a cancer journal for clinicians* 2015, 65(2):87-108.
2. Ferlay J, Soerjomataram I, Dikshit R, Eser S, Mathers C, Rebelo M, Parkin DM, Forman D, Bray F: Cancer incidence and mortality worldwide: Sources, methods and major patterns in GLOBOCAN 2012. *International Journal of Cancer* 2015, 136(5):E359-E386.
3. Bruni L, Barrionuevo-Rosas L, Albero G, Aldea M, Serrano B, Mena M, Gómez D, Muñoz J, Bosch F, de Sanjosé S: Human Papillomavirus and Related Diseases in Uganda. Summary Report 2016. In. Barcelona, Spain: ICO Information Centre on HPV and Cancer (HPV Information Centre).
4. World Health Organization: Comprehensive Cervical Cancer Control: A Guide to Essential Practice. 2nd edition. In. Geneva: World Health Organization; 2014.
5. Twinomujuni C, Nuwaha F, Babirye JN: Understanding the Low Level of Cervical Cancer Screening in Masaka Uganda Using the ASE Model: A Community-Based Survey. *PloS one* 2015, 10(6):e0128498.
6. Ndejjo R, Mukama T, Musabyimana A, Musoke D: Uptake of Cervical Cancer Screening and Associated Factors among Women in Rural Uganda: A Cross Sectional Study. *PLoS ONE* 2016, 11(2):e0149696.
7. Mutyaba T, Mmiro FA, Weiderpass E: Knowledge, attitudes and practices on cervical cancer screening among the medical workers of Mulago Hospital, Uganda. *BMC medical education* 2006, 6(1):13.
8. Mwaka AD, Okello ES, Wabinga H, Walter FM: Symptomatic presentation with cervical cancer in Uganda: a qualitative study assessing the pathways to diagnosis in a low-income country. *BMC women's health* 2015, 15(1):1.
9. Mwaka AD, Okello ES, Kiguli J, Rutebemberwa E: Understanding cervical cancer: an exploration of lay perceptions, beliefs and knowledge about cervical cancer among the Acholi in northern Uganda. *BMC women's health* 2014, 14(1):1.
10. Mutyaba T, Faxelid E, Mirembe F, Weiderpass E: Influences on uptake of reproductive health services in Nsangi community of Uganda and their implications for cervical cancer screening. *Reprod Health* 2007, 4(4).

For peer review only - http://bmjopen.bmj.com/site/about/guidelines.xhtml

11. Paul P, Winkler JL, Bartolini RM, Penny ME, Huong TT, Kumakech E, Mugisha E, Jeronimo J: Screen-and-treat approach to cervical cancer prevention using visual inspection with acetic acid and cryotherapy: experiences, perceptions, and beliefs from demonstration projects in Peru, Uganda, and Vietnam. *The oncologist* 2013, 18(12):1278-1284.

12. Teng FF, Mitchell SM, Sekikubo M, Biryabarema C, Byamugisha JK, Steinberg M, Money DM, Ogilvie GS: Understanding the role of embarrassment in gynaecological screening: a qualitative study from the ASPIRE cervical cancer screening project in Uganda. *BMJ open* 2014, 4(4):e004783.

13. Were E, Nyaberi Z, Buziba N: Perceptions of risk and barriers to cervical cancer screening at Moi Teaching and Referral Hospital (MTRH), Eldoret, Kenya. *African health sciences* 2011, 11(1).

14. Mwaka AD, Wabinga HR, Mayanja-Kizza H: Mind the gaps: a qualitative study of perceptions of healthcare professionals on challenges and proposed remedies for cervical cancer help-seeking in post conflict northern Uganda. *BMC family practice* 2013, 14(1):1.

15. Uganda Bureau of Statistics: The National Population and Housing Census 2014 – Main Report

In. Kampala: Uganda Bureau of Statistics; 2016.

16. Hsieh H-F, Shannon SE: Three approaches to qualitative content analysis. *Qualitative health research* 2005, 15(9):1277-1288.

17. Tong A, Sainsbury P, Craig J: Consolidated criteria for reporting qualitative research (COREQ): a 32-item checklist for interviews and focus groups. *International journal for quality in health care* 2007, 19(6):349-357.

18. Mukama T, Ndejjo R, Musabyimana A, Halage AA, Musoke D: Women's knowledge and attitudes towards cervical cancer prevention: a cross sectional study in Eastern Uganda. *BMC Women's Health* 2017, 17(1):9.

19. Hasahya OT, Berggren V, Sematimba D, Nabirye RC, Kumakech E: Beliefs, perceptions and health-seeking behaviours in relation to cervical cancer: a qualitative study among women in Uganda following completion of an HPV vaccination campaign. *Global health action* 2016, 9.

For peer review only - http://bmjopen.bmj.com/site/about/guidelines.xhtml

20. Ndejjo R, Mukama T, Musinguzi G, Halage AA, Ssempebwa JC, Musoke D: Women's intention to screen and willingness to vaccinate their daughters against cervical cancer – A cross sectional study in eastern Uganda. *BMC Public Health* 2016.

21. Mingo AM, Panozzo CA, DiAngi YT, Smith JS, Steenhoff AP, Ramogola-Masire D, Brewer NT: Cervical cancer awareness and screening in Botswana. *International journal of gynecological cancer: official journal of the International Gynecological Cancer Society* 2012, 22(4):638.

22. Ngugi CW, Boga H, Muigai AW, Wanzala P, Mbithi JN: Factors affecting uptake of cervical cancer early detection measures among women in Thika, Kenya. *Health care for women international* 2012, 33(7):595-613.

23. Singh P, Sachs JD: 1 million community health workers in sub-Saharan Africa by 2015. *The Lancet* 2013, 382(9889):363-365.

24. Bhutta ZA, Lassi ZS, Pariyo G, Huicho L: Global experience of community health workers for delivery of health related millennium development goals: a systematic review, country case studies, and recommendations for integration into national health systems. *Global Health Workforce Alliance* 2010, 1:249-261.

25. Elliott PF, Belinson SE, Ottolenghi E, Smyth K, Belinson JL: Community health workers, social support and cervical cancer screening among high-risk groups in rural Mexico. *Journal of health care for the poor and underserved* 2013, 24(4):1448-1459.

26. Agurto I, Arrossi S, White S, Coffey P, Dzuba I, Bingham A, Bradley J, Lewis R: Involving the community in cervical cancer prevention programs. *International Journal of Gynecology & Obstetrics* 2005, 89:S38-S45.

27. Bukirwa A, Mutyoba JN, N.Mukasa B, Karamagi Y, Odiit M, Kawuma E, Wanyenze RK: Motivations and barriers to cervical cancer screening among HIV infected women in HIV care: a qualitative study. *BMC Women's Health* 2015, 15(1):1-11.

28. Kumakech E, Andersson S, Wabinga H, Berggren V: Integration of HIV and cervical cancer screening perceptions and preferences of communities in Uganda. *BMC women's health* 2015, 15(1):1.

29. Kumakech E, Andersson S, Wabinga H, Berggren V: Integration of HIV and cervical cancer screening perceptions of healthcare providers and policy makers in Uganda. *BMC public health* 2014, 14(1):1.

For peer review only - http://bmjopen.bmj.com/site/about/guidelines.xhtml

Research checklist: Consolidated criteria for reporting qualitative research (COREQ): a 32-item checklist for interviews and focus groups

No	Item	Guide questions/description	Comment	Page number
**Domain 1: Research team and reflexivity**				
Personal Characteristics				
1.	Interviewer/facilitator	Which author/s conducted the interview or focus group?	FGD interviews conducted by Research Assistants with experience in qualitative research and native speakers of the language. KI interviews were conducted by two members of the research team.	Page 6
2.	Credentials	What were the researcher's credentials? *E.g. PhD, MD*	RN – BEHS, MSc (Environmental Health Scientist) TM – BEHS, MPH, (Public Health Specialist) JK – BA(SS), PGDE, MDS, PhD (Anthropologist) DM – BEHS, MSc, PhD (Public Health Specialist)	Methods – page 6
3.	Occupation	What was their occupation at the time of the study?	All authors are Public Health Researchers and university lecturers.	N/A
4.	Gender	Was the researcher male or female?	Male researchers conducted the KIs. FGDs were conducted by female Research Assistants.	
5.	Experience and	What experience or	All authors had	

For peer review only

	training	training did the researcher have?	conducted qualitative research and published qualitative papers.	
Relationship with participants				
6.	Relationship established	Was a relationship established prior to study commencement?	No relationship was established with research participants before the study.	N/A
7.	Participant knowledge of the interviewer	What did the participants know about the researcher? e.g. *personal goals, reasons for doing the research*	The participants were informed about the research project, its objectives and purpose and why they were selected to participate. The study also had ethical approval which provided the names and contact of the first author.	Methods – page 7
8.	Interviewer characteristics	What characteristics were reported about the interviewer/facilitator? e.g. *Bias, assumptions, reasons and interests in the research topic*	No interviewer-related biases were identified or reported.	N/A
**Domain 2: study design**				
Theoretical framework				
9.	Methodological orientation and Theory	What methodological orientation was stated to underpin the study? *e.g. grounded theory, discourse analysis, ethnography, phenomenology, content analysis*	Directed content analysis	Page 6 & 7
Participant selection				
10.	Sampling	How were participants selected? *e.g. purposive, convenience, consecutive, snowball*	FGD participants were selected through purposive sampling with	Methods – page 5

For peer review only

			guidance from local leaders and community health workers. Similarly, KIs were selected purposively due to their knowledge and influence.	
11.	Method of approach	How were participants approached? e.g. *face-to-face, telephone, mail, email*	FGD participants were approached face to face while KIs were reached through the phone.	Methods – page 6
12.	Sample size	How many participants were in the study?	119 screening-eligible women participated in the FGDs. There were also 11 KIs.	Results – page 8 and 5.
13.	Non-participation	How many people refused to participate or dropped out? Reasons?	No participant either refused to participate or dropped out	Results – page 8
Setting				
14.	Setting of data collection	Where was the data collected? e.g. *home, clinic, workplace*	FGDs were conducted in community gathering places identified by the research team in collaboration with local leaders. On the other hand, KIs were conducted mostly at their work places	Methods – page 6
15.	Presence of non-participants	Was anyone else present besides the participants and researchers?	No other person was around except the participants and researchers.	Methods – page 6
16.	Description of sample	What are the important characteristics of the sample? e.g. *demographic data, date*	Detailed description of important characteristics provided in table 1.	Results – page 8
Data collection				
17.	Interview guide	Were questions, prompts, guides provided by the authors? Was it pilot	FGD and KI guides used had questions and prompts provided by the	Methods – page 6

For peer review only

		tested?	authors. However, the interviewers were free to ask additional probing questions.	
18.	Repeat interviews	Were repeat interviews carried out? If yes, how many?	No	N/A
19.	Audio/visual recording	Did the research use audio or visual recording to collect the data?	Audio recordings used during data collection	Methods – page 6
20.	Field notes	Were field notes made during and/or after the interview or focus group?	Field notes were made during the interview	Methods – page 6
21.	Duration	What was the duration of the interviews or focus group?	FGDs lasted about 1 hour while KIs lasted about 45 minutes.	Methods – page 6
22.	Data saturation	Was data saturation discussed?	Yes	Methods – page 5
23.	Transcripts returned	Were transcripts returned to participants for comment and/or correction?	No	N/A
**Domain 3: analysis and findings**				
Data analysis				
24.	Number of data coders	How many data coders coded the data?	2	Methods – page 6
25.	Description of the coding tree	Did authors provide a description of the coding tree?	Coding process described	Methods – page 6 & 7
26.	Derivation of themes	Were themes identified in advance or derived from the data?	Some themes were identified in advance while others arose the data.	Methods – page 6
27.	Software	What software, if applicable, was used to manage the data?	Atlas ti version 6.0.15	Methods – page 7
28.	Participant checking	Did participants provide feedback on the findings?	No	N/A
Reporting				
29.	Quotations presented	Were participant quotations presented to	Quotations are presented to	Results – pages 9-16

For peer review only

		illustrate the themes / findings? Was each quotation identified? e.g. *participant number*	highlight themes and participants anonymized by FGD number, participant number and age.	
30.	Data and findings consistent	Was there consistency between the data presented and the findings?	Yes	Results – pages 9-16
31.	Clarity of major themes	Were major themes clearly presented in the findings?	Yes	Results – pages 9-16
32.	Clarity of minor themes	Is there a description of diverse cases or discussion of minor themes?	Yes, discussion of minor themes provided within manuscript	Results – pages 9-16 and discussion – pages 17-18

For peer review only
